# Relationship between Diabetes with Drug Use, Family History and Alcohol; Insights from the PERSIAN Cohort Study for Health Service Management -

**DOI:** 10.31661/gmj.v14i.3853

**Published:** 2025-12-27

**Authors:** Mohammad Khammarnia, Alireza Ansari-Moghaddam, Zahra Takamoli Poshtehee, Fariba Shahraki-Sanavi, Mahdi Mohammadi

**Affiliations:** ^1^ Health Promotion Research Center, Zahedan University of Medical Sciences, Zahedan, Iran

**Keywords:** Diabetes, Family History, Alcohol, Drug

## Abstract

**Background:**

In recent decades, the prevalence of diabetes, alcohol and tobacco consumption has increased in Iran and the world. This study aimed to investigate the relationship between diabetes with family history, alcohol and tobacco consumption in Iran.

**Materials and Methods:**

A cross-sectional study was carried out in the southeast of Iran in 2023_2024. The target population consisted of 10,016 individuals aged between 35 and 70 years. The data required for this study was obtained from the data collected by ZACS (Zahedan Adult Cohort Study) in the southeast of Iran (The Persian Cohort and ZACS data are the same. In fact, ZACS is part of the Persian Cohort). The data on general information, diabetes, family history of diabetes and alcohol consumption were collected through a standard questionnaire and were analyzed using descriptive statistics and independent t-tests, chi-square and logistic regression in SPSS 22 software.

**Results:**

The prevalence of diabetes was 19.0% and it was more prevalent in women (20.1%), unemployed people (21.9%) and age groups of 60 years and older (P0.05). The prevalence of diabetes was 37.8% in first relatives and 19.4% in second relatives. Although only 2.4% of people consumed alcohol, diabetes was less common in people who consumed alcohol than in others (P0.001). In univariate results, drug use reduced the chance of developing diabetes (OR=0.86, P=0.026). However, there was not significant relationship between drug use and diabetes after adjusting for history of diabetes in relatives (OR=0.99, P=0.229). Family history had a positive effect on developing diabetes (P0.001).

**Conclusion:**

The findings showed the family history had a positive effect on developing diabetes. These results show the necessity of examining the family history of people, identifying people at risk, and also providing the necessary education for the prevention of diabetes. It is recommended that people with a family history of diabetes take diabetes preventive measures and modify their lifestyle.

## Introduction

Diabetes is one of the types of chronic diseases in which abnormally high levels of blood glucose are observed [1]. Globally, diabetes is the leading non-communicable and chronic disease that has various complications [2] Diabetes is growing in developing and developed countries, and according to predictions, it will affect about 693 million adults by 2045, which has increased by more than 50% compared to 2017 [2]. Also, the increase of this disease has also turned from the eighth cause of disability to the sixth cause in the world [3]. From a social perspective, diabetes imposes a heavy burden on the health economy during the treatment period and its complications, including mortality [4]. Diabetes causes many physical, psychological and social problems for individuals and society [5]. Also, diabetes has various complications, including: nephropathy, retinopathy, cardiovascular and renal complications [6]. Diabetes caused by certain factors including genetic, pancreatic factors, and others and gestational diabetes. The highest disease burden is related to type 1 and 2 diabetes [7]. Type 1 and 2 diabetes are common diseases that affect a large number of people around the world and lead to negative health effects as well as increased costs in the field of health. In addition, this disease affects the quality of life and reduces it. The risk factors that cause type 2 diabetes are lifestyle and genetics, which interact with each other and people's living environment [8]. Absence of insulin secretion from pancreatic beta cells is a precursor to type 1 diabetes [9]. According to researches, type 2 diabetes and high blood pressure are among the things that cause cardiovascular diseases, and people who suffer from these two diseases at the same time are more likely to die than people who suffer from one of these two diseases and cardiovascular mortality [10]. People's awareness about long-term effects of diabetes and the types of diabetes makes people aware of the importance of prevention [11]. Increased survival in the elderly as well as increased obesity and sedentary lifestyle in all age groups are important factors for more people to develop type 2 diabetes [12]. Cardiovascular risk factors in type 2 diabetes that are preventable include smoking, hypertension, and potentially hyperglycemia. [13]. Alcohol is one of the natural and pain-relieving substances that gives a person a euphoric feeling. This substance causes poisoning and also suppresses brain function. The negative effects of alcohol are not specific to the individual, but also affect the family and society [14]. In South Asia, where there are many types of tobacco, this substance, along with the problematic consumption of alcohol, leads to many diseases and deaths [15]. Given that alcohol and drug use are two factors that contribute to the development of diseases, it is necessary to study the relationship between these two factors and diabetes. Considering the high statistics of diabetes and smoking and alcohol consumption in the world and Iran, this study was conducted with the aim of investigating the relationship between diabetes with family history, alcohol and smoking consumption.

## Materials and Methods

### Study Design

This is a cross-sectional study conducted on adult population aged between 35 to 70 years in Zahedan, southeast of Iran. The data were extracted from the enrolment phase of Zahedan cohort adults (ZACS) conducted between 2014 and 2019 [16].

Zahedan, the capital of the country's second largest province, is located in the southeast of Iran with a population of more three million people and has a border of 1100 kilometers with Afghanistan and Pakistan [17]. This province mainly consists of Baluch and Sistani ethnicity. This province has the lowest economic growth rate among the provinces of the country due to cultural factors, geographical location, and also environmental factors.

**Figure-1:**
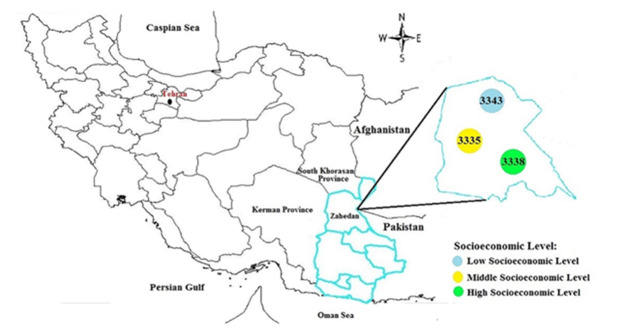
Figure 1. Geographical location of Zahedan and division into three regions based on socioeconomic status

Figure-1, shows the geographical location of Zahedan in southeastern Iran and the spatial distribution of baseline populations in the Zahedan Adult Cohort Study.

Adults with at least 9 months of residence in Zahedan or immigrants from other regions with at least one year of residence were included in the study. Written informed consent was obtained from the participants. The participants in this study were excluded from the study if they did not follow the study rules or were suffering from physical and mental diseases or were unable to answer the questionnaires and referring to the cohort center due to their conditions. A multi-stage random sampling method was used to select the participants randomly. First, study population was divided into 3 regions based on socioeconomic status and a central health center was determined in the regions. A sample of 10016 people was selected randomly from the population covered by those health centers [18] .

### Ethics Approval

The ethics committee of Zahedan University of Medical Sciences approved the study protocol (approval numbers: IR.ZAUMS.REC. 1402.247). This investigation was in accordance with the Declaration of Helsinki of 1975. Additionally, a written informed consent form was obtained from all participants.

### Statistical Analysis

All statistical analyses were performed using SPSS 22 software (IBM SPSS Statistics for Windows, Armonk, New York, USA). For descriptive analysis, mean and standard deviation (SD) were used for quantitative variables, and frequency distribution for qualitative variables. Chi-square test was used to compare diabetes and non-diabetes based on demographic, history of diabetes, and substance use. Relationship between drug use and diabetes was adjusted for possible confounding factors using logistic regression model. The significant level was set to 0.05.

## Result

**Table:** Table1. Demographic and Descriptive Factors Affecting the History of Diabetes among Participant in Southeast of Iran

**Variables**	**Dimension**	**Total** **n= 10016**	**Non-Diabetes n= 8109 **	**Diabetics N (%) n= 1907 **	**P-value**
		**n(%)**	**n(%)/Mean±SD**	**n (%)/Mean±SD**	
	35-40	1877(18.7)	1785(95.1)	92(4.9)	
	41-45	1524(15.2)	1785(95.1)	157(10.3)	
**Age (year)**	46-50	1609(16.1)	1357(84.3)	252(15.7)	<0.001
	51-55	1764(17.6)	1330(75.4)	434(24.6)	
	56-60	1663(16.6)	1194(71.8)	469(28.2)	
>60	1579(15.8)	1076(68.1)	503(31.9)
**Gender**	Male	3917(39.1)	3237 (82.6)	680(17.4)	0.001
	Female	6099(60.9)	4872 (79.9)	1227(20.1)	
**Has job**	No	7056(70.4)	5510(78.1)	1546(21.9)	<0.001
	Yes	2960(29.6)	2599(87.8)	361(12.2)	
**History of diabetes in first relatives **	No	6226(62.2)	5414(87.0)	812(13.0)	<0.001
	Yes	3789(37.8)	2694(71.1)	1095(28.9)	
**History of diabetes in second relatives **	No	8076(80.6)	6599(81.7)	1477(18.3)	<0.001
	Yes	1939(19.4)	1509(77.8)	430(22.2)	
**Use** **alcohol**	No	9779(97.6)	7898(80.8)	1881(19.2)	0.001
	Yes	236(2.4)	210(89.0)	26(11.0)	
**Current Smoking **	No	9451(96.6)	7601(80.4)	1850(19.6)	<0.001
	yes	565(5.6)	508(89.9)	57(10.1)	
**Use drugs**	No	8112(81.0)	6533(80.5)	1579(19.5)	0.026
	Yes	1903(19.0)	1575(82.8)	328(17.2)	
	Opium	1008(10.1)	805(79.9)	203(20.1)	
	Shireh	739(7.4)	639(86.5)	100(13.5)	
	Other	8(0.1)	8(100.0)	0(0.0)	
	Change opium to shireh	20(0.2)	15(75.0)	5(25.0)	
Drug Type ^*^	Change shireh to opium	85(0.8)	69(81.2)	16(18.8)	0.001
	Change others to others	29(0.3)	28(96.6)	1(3.4)	
Drug Use Type ^*^	Oral	463(4.6)	387(83.6)	76(16.4)	0.600
	Inhalation	1425(14.2)	1176(82.5)	249(17.5)	
**Duration of** Drug Use^*^(year)			15.04±10.98	13.30±12.65	0.012

^*^ Missing data

**Table:** Table2. Relationship between Diabetes and Drug use based on Logistic Regression among Participant in Southeast of Iran

	**Adjustment**	**OR (95% CI)**	**P-value**
**Model 1**	None	0.86 (0.76- 0.98)	0.026
**Model 2**	Gender, Age and Job	0.77(0.67-0.89)	<0.001
**Model 3**	First relatives & Second relatives	0.92(0.80-1.05)	0.229

Logistic Regression (model 1): Use Drugs Logistic Regression (model 2): Use Drugs with Gender ID, Age and Job Logistic Regression (model 3): Use Drugs and First relatives & Second relatives

A total of 10016 adults participated in the study. Half of the participants were older than 50 years, 60.9% were female, and 29.6% were employed. Among all, 5.6% were cigarette-smoker, 2.4% were alcohol user, and 19% were drug user. The prevalence of diabetes was 37.8% in first relatives and 19.4% in second relatives (Table-1).

The prevalence of diabetes was 19.0% and it was significantly higher in participants with a family history of diabetes (P<0.001). Diabetes was more prevalent in Women (20.1%) than men (17.4%) (P=0.001). The prevalence of diabetes increased from 4.9% in individuals aged 35-40 years to 31.9% in participants older than 60 years (P<0.001). The prevalence of diabetes was lower in employed people (P<0.001), cigarette smokers (P<0.001), drug users (P=0.026), and alcohol users (P=0.001). The type of drug use was mostly inhalation (17.5%) with no significant relationship between the type of drug use and diabetes (P=0.600). Among drug users, 1564 (82.8%) were non-diabetes and 325 (17.2%) were diabetes and the average duration of drug use was longer in people who did not have diabetes (P=0.012, Table-1). According to Table-2, drug use decreased the odds of diabetes significantly (OR=0.86, P=0.026). The odds of diabetes in drug users decreased by 23% after adjusting for demographic factors (gender, age, and job) (OR=0.77, P<0.001). However, there was not significant relationship between drug use and diabetes after adjusting for history of diabetes in relatives (OR=0.99, P=0.229). The family relationship variable, as a main variable, has a great impact on diabetes, so that with the presence of this variable, the impact of tobacco use variable becomes weak or ineffective.

## Discussion

This study provides an estimate of the association between diabetes with family history and alcohol and drug use.

The results of this study showed that there is a relationship between family history and diabetes. So, people who have a family member with diabetes are more likely to develop this disease. Consist with this finding, another study also shows a relationship between family history of diabetes and the number of relatives with diabetes[19]. Han et al. indicated that the gene score factor has been identified as a risk factor for diabetes[20]. Genetics alone is not the cause of diabetes, but other factors also play a role in diabetes. Genetic predisposition to diabetes increases the chance of developing it. The findings of another study show that diabetic foot complications are among the complications that patients with type 2 diabetes have a clear relationship with family history (first-degree family with diabetes). Other studies show that the risk factors of this disease include smoking, gender [21] . Ansari Moghadam et al showed that the annual treatment cost of diabetic patients with a family history of diabetes is higher more than other patients [3]. Therefore, in families with a family history of diabetes, family members should take more care and preventive measures to prevent the disease.

According to the findings of this study, although people who used drugs and alcohol were less likely to develop diabetes, based on regression, after controlling for the effect of family relationships, there was no relationship between drug use and alcohol with diabetes. The results obtained in the study conducted in Golestan indicate that people with diabetes who use opioids have a shorter life compared to people with diabetes who do not use them [22] . Sistan and Baluchestan Province is one of the border provinces of the country that borders drug-producing countries. For this reason, access to drugs is easier and their prevalence is higher in this province [23]. This province also has the highest number of addiction cases [24]. A study conducted by Winhusen et al shows that people who consume alcohol die more often than people with diabetic neuropathy. Diabetic people who consume alcohol are more likely to develop neuropathy [25]. Another study shows that drinking alcohol reduces the risk of diabetes and smoking increases the risk of type 2 diabetes [26].

According to the findings, diabetes is more common in women. But in another study, it has been shown that the probability of type 2 diabetes is higher in men than in women [27]. Sex hormones have an effect on diabetes and the beneficial effect of these hormones is more in women than in men [28]. Steroid hormones play a protective role in premenopausal women and reduce the risk of diabetes [29].

According to the results, there is the relationship between diabetes and age, so with increasing age, the probability of diabetes increases. Another study also shows that elderly people, especially people over 40 years old and smokers and alcohol users, are affected more than other people [27]. Khamarnia et al.'s study also showed that type 2 diabetes is more common in the age group of 60 years and older [5]. Elderly people are more likely to develop diabetes than other age groups due to lack of exercise, lifestyle, and improper diet. Therefore, the elderly is a group at risk of diabetes and require more attention to prevent and control the disease.

This study showed in univariate results that smoking reduces the incidence of diabetes. Contrary to the findings of this study, Alamo and et.al stated in their study that smoking causes diabetes by increasing the body's resistance to insulin, and this issue requires further investigation [30]. The findings of another study show that the risk factors of this disease include smoking [21]. Ivan Berlin et al. showed in their study that starting smoking before developing diabetes is considered a risk factor for diabetes, and starting smoking after developing diabetes causes poor blood sugar control [31].

## Conclusion

The findings indicate that a positive family history significantly increases the risk of developing diabetes. These results underscore the importance of incorporating family history assessments into routine health evaluations and screening programs. From a health services management perspective, identifying individuals at high risk allows for targeted preventive interventions, efficient allocation of resources, and implementation of educational programs promoting lifestyle modification. Integrating such strategies within primary care and community health services can enhance early detection, patient engagement, and the overall effectiveness of diabetes prevention efforts, ultimately improving health outcomes and supporting more sustainable health service delivery.

## Conflict of Interest

The authors have declared that no competing interests exist
